# Patient Perceptions of Provider Race Concordance and Quality of Chronic Illness Care

**DOI:** 10.1007/s11606-024-09025-w

**Published:** 2025-01-22

**Authors:** Benjamin Grant, Orysya Soroka, Elizabeth Baquero, Joanna Bryan Ringel, Andrea Cherrington, Doyle M. Cummings, Jacqueline R. Halladay, Arvind Rajan, Monika M. Safford

**Affiliations:** 1 Weill Cornell Medical College, Weill Cornell Division of General Internal Medicine, New York, NY USA; 2 The University of Alabama at Birmingham, Birmingham, AL USA; 3 East Carolina University, Greenville, NC USA; 4 The University of North Carolina at Chapel Hill, Chapel Hill, NC USA

**Keywords:** race, concordance, chronic illness, hypertension, discordance

## Abstract

**Background:**

Black people are more likely to have hypertension and report lower quality of care than White people. Patient-provider race concordance could improve perceived quality of care, potentially lessening disparities.

**Objective:**

Investigate the association between patient-provider race concordance and patient-perceived quality of chronic disease care, as measured by the Patient Assessment of Chronic Illness Care (PACIC) scale.

**Design:**

Cross-sectional analysis of baseline data from a randomized trial with Black patients with persistently uncontrolled hypertension.

**Setting:**

Participants received care at one of 69 rural primary care practices in Alabama and North Carolina.

**Participants:**

Three hundred and ninety-one Black patients with persistently uncontrolled hypertension enrolled in the Southeastern Collaboration to Improve Blood Pressure Control (SEC) trial.

**Main Measure:**

PACIC overall scores and subscale scores (*patient activation*, *delivery system*, *goal setting, problem solving*, *follow-up*).

**Results:**

Of 1592 patients enrolled in the SEC trial, 391 participants self-reported race concordance data and completed the PACIC. Most participants were age < 60 (52.4%), 65.2% identified as women, and 50.1% were beneficiaries of either Medicare or Medicaid. Those with patient-provider race concordance reported higher overall PACIC scores (58.8% vs 46.1%, *p* < 0.05), with higher sub-scores of goal setting (60.9% vs 46.8%, *p* < 0.05) and problem-solving (62.7% vs 48.0%,* p* < 0.05) compared to those without race concordance. Poisson regression models of participants age ≥ 60 years demonstrated that those with race concordance were more likely to have higher overall PACIC scores (RR 1.53, 95% CI 1.17–2.0, *p* = 0.002), goal-setting subscale scores (RR 1.63, 95% CI 1.24–2.15, *p* = 0.0005), and problem-solving subscale scores (RR 1.66, 95% CI 1.29–2.14, *p* < 0.0001). Those < 60 years of age had no significant findings comparing those with and without race concordance.

**Conclusions:**

Older Black patients perceived greater quality of care if their providers were also Black.

**Visual abstract:**

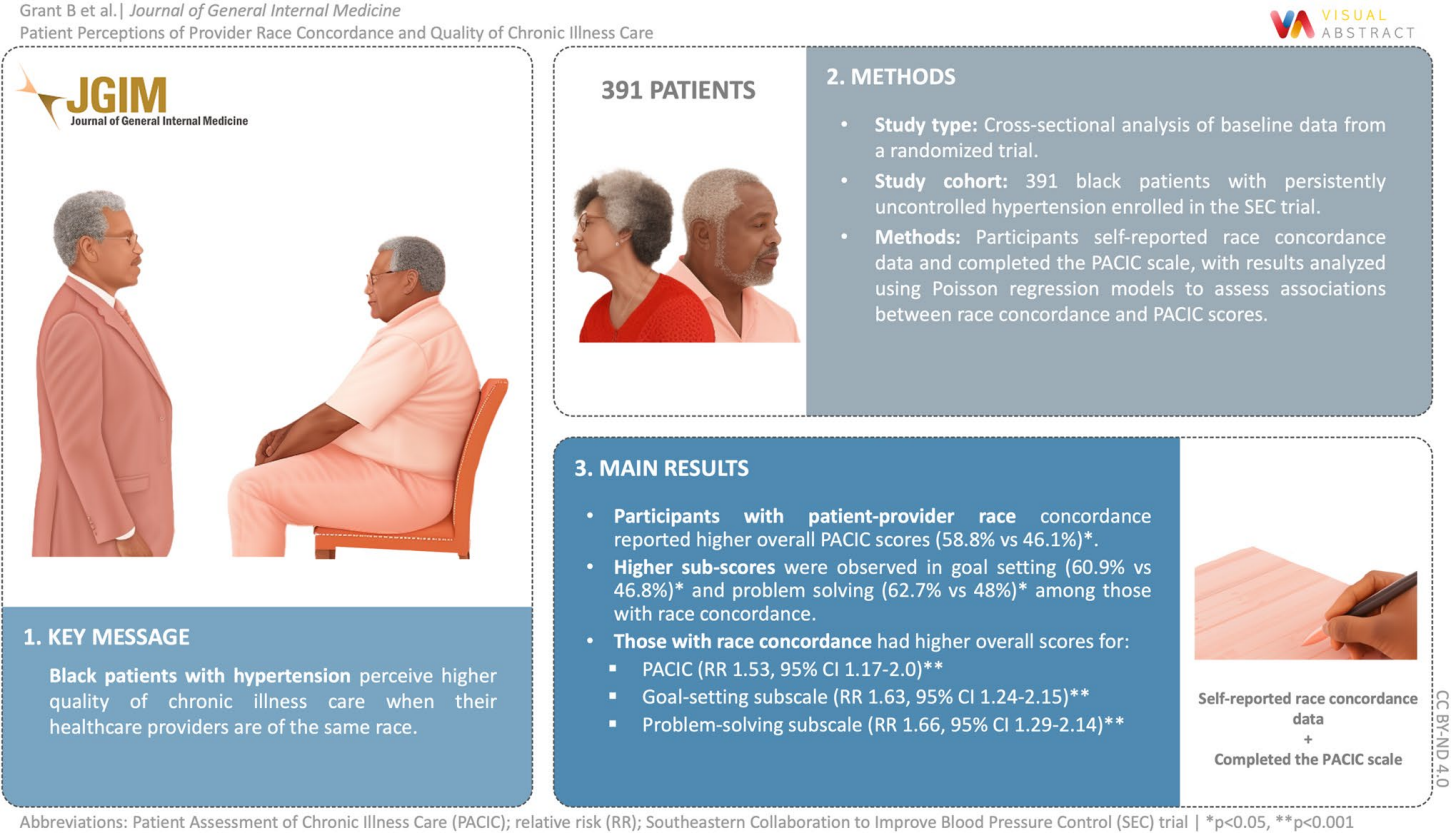

**Supplementary Information:**

The online version contains supplementary material available at 10.1007/s11606-024-09025-w.

## BACKGROUND

In the USA, Black patients with hypertension (HTN) experience startling healthcare disparities. While the national prevalence of HTN among adults is 45%, the prevalence of HTN in Black adults is 57% while that of White adults is 43%.^1^ Not only are Black individuals more likely to have HTN, they are 57% more likely to die of its complications compared to White individuals.^2–4^ Such disparities have contributed to medical mistrust in Black communities, poor follow-up, and Black patients perceiving their care as lower quality than the care provided to White patients.^5^

To improve outcomes and equitable care for Black patients, it is important to identify possible drivers of these disparities. Individual characteristics like race, ethnicity, socioeconomic status, and geographic location are associated with disparities in patient care experiences and outcomes.^6^ The patient-provider relationship, defined as an interaction in which healthcare professionals serve the medical needs of patients, has been shown to have a significant impact on healthcare disparities.^7^ Previous studies have suggested that patient-provider relationships may be strengthened, and these disparities reduced, in race-concordant patient-physician pairings (defined as a patient and physician sharing the same racial identity) as evidenced by increased uptake of preventive services and increased levels of patient trust and intent to adhere to care plans compared to race-discordant pairings.^5,7, 8^ Patients in race-concordant clinical interactions are more likely to have higher health literacy, increased preventative healthcare, more therapeutic relationships with their providers, and are associated with better blood pressure control.^7–11^ Furthermore, studies have demonstrated that Black patients in race-discordant clinical interactions report poorer quality of care.^5^

While studies have demonstrated an association between race concordance and patient-perceived quality of care, no studies, to our knowledge, have quantitatively measured this relationship in the rural Southeastern US, a region with strikingly poor health outcomes. We hypothesized that Black patients with uncontrolled HTN who perceive racial concordance with their provider will report higher quality chronic disease care as captured using the Patient Assessment of Chronic Illness Care (PACIC), compared to those in race discordant pairings.

We examined this hypothesis in the context of the Southeastern Collaboration to Improve Blood Pressure Control (SEC), a trial of exclusively Black patients with persistently uncontrolled HTN residing in the southeastern US Black Belt region. This population is at high risk for poor outcomes, heightening the importance of excellent care of their chronic illness. Additionally, because older adults have a larger burden of chronic diseases, we examined differences by age.^12^ This unique population has historically been overlooked in the literature, and as such, gaps in our understanding of the needs and issues affecting them persist.

Importantly, we examined our hypothesis within the framework of the Chronic Care Model (CCM). Developed in 1998, Wagner’s CCM has become the standard for high-quality chronic illness care.^13,14^ Building on Wagner’s work, the National Committee on Quality Assurance has offered primary care practices Patient Centered Medical Home (PCMH) recognition, indicating that elements of the CCM have been implemented. The PACIC is a tool used to capture how well the CCM is implemented at the practice based on a patient’s experience. We selected the PACIC as it is validated and associated with positive self-management behaviors (i.e., medication adherence) which can lead to improved health outcomes.^15^

Furthermore, the PACIC includes subscales that measure domains of the CCM: *Patient Activation*, *Delivery System Design*, *Goal Setting*, *Problem-Solving*, and *Follow-Up*. In primary care, these aspects of clinic visits are important for promoting behavioral change in patients and improving their outcomes.^14^ *Patient Activation* measures how often patients were given opportunities to collaborate with their provider when determining their care plan and is strongly associated with health outcomes.^16^ *Delivery System Design* refers to the structure and organization of a patient’s healthcare system, and focuses on providing holistic care.^17^ *Goal Setting* involves patients and providers collaboratively agreeing on a course of actionable steps that move a patient towards a specific health-related goal.^18^ *Goal Setting* can encourage positive lifestyle changes as evidenced by healthier food choices, better medication adherence, and better disease control.^19–22^ *Problem-solving* measures how well a provider was perceived to use a patient-centered approach to develop a care plan that considers the patient’s needs, values, and preferences.^23^ *Follow-Up* reflects how well a patient’s needs were attended to at the conclusion of a visit. In this study, we analyzed associations between race concordance and the PACIC overall and subscale scores, overall, and in participants age < 60 and ≥ 60 years.

## METHODS

### Design

This cross-sectional study used data collected at baseline from a subsample of participants in the SEC cluster randomized trial. Details of the trial are provided elsewhere.^24^ Briefly, all 1592 participants self-identified as Black and all had persistently uncontrolled HTN, defined as mean clinic systolic blood pressure (BP) ≥ 140 mm Hg in the year prior to enrollment, plus BP at the time of enrollment assessed by a research assistant ≥ 140/90 mm Hg. Participants received primary care from one of 69 practices in Alabama and North Carolina.^24^ SEC investigators tested the effectiveness of peer coaching and practice facilitation on improving BP and BP control. The protocol was approved by institutional review boards at participating universities; all participants provided written informed consent (clinicaltrials.gov NCT02866669).

### Setting/Study Sample

The study sample included only participants who completed both the PACIC and the patient-provider race concordance question at baseline. Investigators added this question after recruitment had been initiated.

### Exposure Variable: Patient-Provider Race Concordance

Participants who responded “yes” to the question “As far as you know, is the primary care provider that you see at this facility the same race or ethnicity as you?” were considered to have race concordance with their provider, and those who responded “no” were considered to have race discordance.

### Main Outcome Variable: Patient Assessment of Chronic Illness Care

At baseline, patients were asked to think back to their most recent clinical encounter and answer questions in the PACIC. The PACIC was developed as a patient-centered method of measuring implementation of the CCM.^15^ The CCM focuses on six key areas essential to high-quality chronic disease management: self-management support, decision support, delivery system design, clinical information systems, organization of healthcare, and community resources. The PACIC is a 20-item questionnaire with responses using a Likert scale comprised of “none of the time,” “a little of the time,” “some of the time,” “most of the time,” or “always” (Supplemental Item 1). The PACIC is validated and has been demonstrated to be both reliable and practical in assessing patient perceived quality of chronic illness care.^15,25^

### Secondary Outcome Variables: PACIC Subscales

The PACIC’s five subscales measure subdomains of the CCM: Patient Activation (items 1–3), Delivery System Design/Decision Support (items 4–6), Goal Setting (items 7–11), Problem-solving/Contextual Counseling (items 12–15), and Follow-up/Coordination (16–20). Of note, the CCM itself consists of six components of patient care and the PACIC subscales do not map perfectly onto these components as most patients are unable to report on less apparent components of their healthcare such as “clinical information systems” and “organization of health care.”

### Other Variables

Additional variables were used to describe the sample and adjust the multivariable analysis. Age and gender were self-reported. Educational attainment was categorized as < high school education vs. high school or more education. Practices self-reported whether they had achieved patient-centered medical home status, and whether they were a Federally Qualified Health Center. We grouped Federally Qualified Health Centers with free and community health center clinics, since all accept patients without health insurance.

### Statistical Analysis

We first compared baseline characteristics of patients who perceived race concordance with their provider with those who did not. We then examined the distribution of PACIC responses. Responses were scored from 0 to 4 with “none of the time” receiving a score of 0, “a little of the time” receiving a score of 1, and so on, with “always” receiving a score of 4. Final PACIC scores were averaged over the 20 questions generating a score range from 0 to 4 for each participant.

We examined quartiles of PACIC scores and compared this analysis to dichotomization of the data at the median. Both methods produced similar results. Due to our modest sample size, the non-normal distribution of scores, and to facilitate interpretation of the findings, dichotomization of the data at the median was used for analyses. PACIC scores ≥ median were classified as better perceived CCM implementation and scores < median were considered to be worse perceived CCM implementation.

We analyzed associations between race concordance vs. discordance and high vs. low PACIC scores by first examining bivariate associations using the chi-square statistic. Since the outcome was common, multivariable models used modified Poisson regression with robust error variance, entering patient characteristics to understand factors associated with higher PACIC scores, and to understand the role of race concordance, independent of these other factors.^26^ Adjusted relative risk (RR) and 95% confidence intervals (CI) were calculated.

To test the hypothesis that differences in having higher PACIC scores between race concordant and race discordant patients may vary across age, we examined interactions between race concordance and age < 60 and ≥ 60 years in an unadjusted model and noted the *p*-value of the interaction term; *p* < 0.15 supported stratification. An important goal of the parent SEC study was to examine the heterogeneity of treatment effects in specific high-risk subgroups. Since younger patients are at higher risk for uncontrolled HTN, the study aimed to recruit approximately equal groups age < 60 and ≥ 60 years. As such, we used age < 60 and age ≥ 60 to dichotomize our data.

We repeated the steps above for each of the five subscales, calculating each subscale score as an average over items in that subscale.

For each of the 20 PACIC questions, three or fewer participants did not provide a valid answer for a total of 19 participants with missing data on the PACIC. We conducted multiple imputation by chained equations to maximize sample size.^27^ These analyses were nearly identical to results using a complete case approach; thus, the complete case analysis results are presented. We performed analyses using SAS statistical software, version 9.4 (SAS Institute, Cary, NC), and Stata, version 14.2 (StataCorp, College Station, TX). A *p*-value of < 0.05 was considered statistically significant.

## RESULTS

### Sample Characteristics

Of the 1592 patients that were a part of the broader SEC study, 391 participants met inclusion criteria for our study. In this sample, 34.8% self-identified as men, 47.6% were age ≥ 60 years, and 36.1% self-reported race concordance with their primary care provider. Characteristics did not differ between groups except that racially concordant pairs were more prevalent in FQHC’s/community clinics (35.5% vs 26.0%, *p* = 0.049) and PCMH-certified facilities (36.1% vs 25.7%, *p* = 0.04) as compared with other healthcare settings (Table 1).

**Table 1:** Characteristics of the 391 Participants, Overall, and by Patient-PCP Race Concordance, from the all-Black SEC (Southeastern Collaboration to Improve Blood Pressure Control) Study

Characteristics	All	Race concordant	Race discordant	*p*-value
*N*	391	141	250	
Age, ≥ 60 years, *n* (%)	186 (47.6%)	71 (50.4%)	115 (46.0%)	0.41
Men, *n* (%)	136 (34.8%)	49 (34.8%)	87 (34.8%)	0.99
Less than high school education, *n* (%)	55 (14.1%)	20 (14.3%)	35 (14.1%)	0.95
Practice PCMH^a^ certified, *n* (%)	107 (29.2%)	44 (36.1%)	63 (25.7%)	0.04
FQHC^b^, *n* (%)	115 (29.4%)	50 (35.5%)	65 (26.0%)	0.049
PACIC^c^ score ≥ median, *n* (%)				
Overall PACIC^c^ score (median = 1.85)	188 (50.5%)	77 (58.8%)	111 (46.1%)	0.02
Patient Activation subscale (median = 1.33)	220 (56.8%)	86 (62.3%)	134 (53.8%)	0.11
Delivery System Design/Decision Support subscale (median = 2.67)	172 (44.3%)	62 (44.3%)	110 (44.4%)	0.99
Goal Setting/Tailoring subscale (median = 1.80)	200 (51.8%)	84 (60.9%)	116 (46.8%)	0.01
Problem-Solving/Contextual subscale (median = 2.50)	203 (53.1%)	84 (62.7%)	119 (48%)	0.01
Follow-up/Coordination subscale (median = 0.80)	220 (56.7%)	88 (62.4%)	132 (53.4%)	0.09

^a^*PCMH*, Patient-centered medical home; ^b^*FQHS*, Federally Qualified Health Center, free clinic, community health center; ^c^*PACIC*, Patient Assessment of Chronic Illness Care

### Overall PACIC Score Findings

**Figure. 1:**
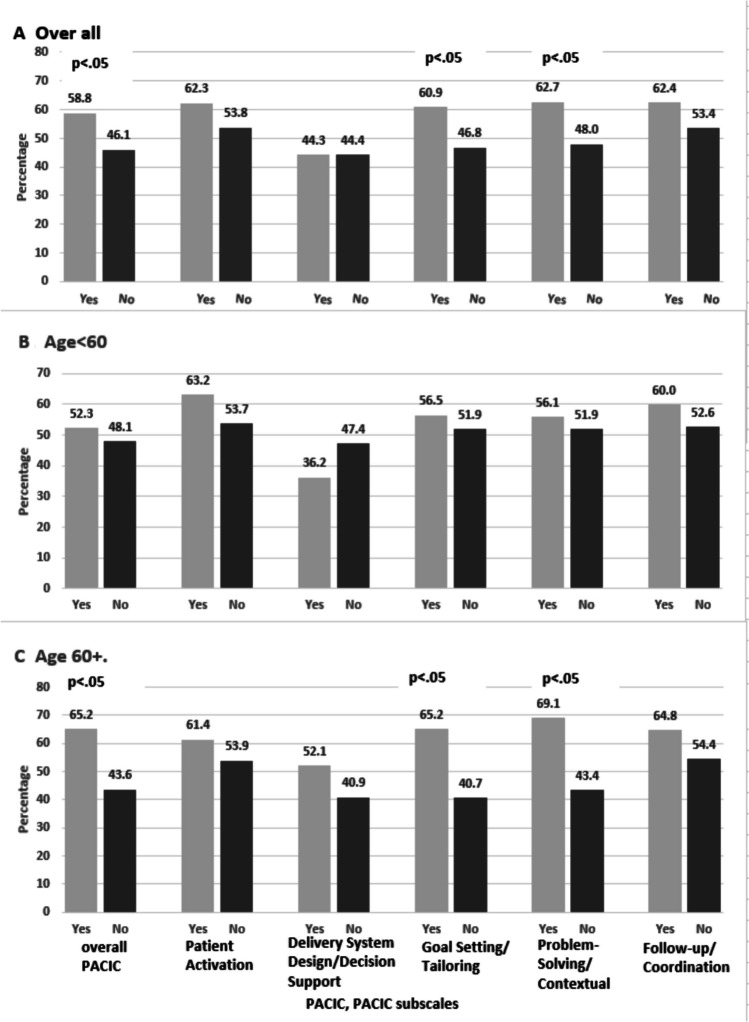
Proportion with below vs. at or above median overall PACIC (Patient Assessment of Care for Chronic Conditions) and subscale scores in race concordant (Yes) and race discordant (No) pairs, overall (**A**) and by age group (**B**, **C**).

A larger proportion of patients in race concordant pairings had higher PACIC scores as compared to their race discordant counterparts (58.8% vs 46.1%, *p* < 0.05) (Fig. 1). Compared to racially discordant pairs, racially concordant pairs were 28% more likely to have high PACIC scores (RR 1.28, 95% CI 1.05–1.56, *p* = 0.016), and these findings continued to be significant when adjusting for age, gender, and highest education level attained (RR 1.29, 95% CI 1.07–1.56, *p* = 0.009) (Table 2).

**Table 2:** Risk Ratios (95% CI (Confidence Interval)) for having Higher PACIC (Patient Assessment of Care for Chronic Conditions) Over and Subscale Scores in Patients with Patient-Provider Race Concordance Versus Patients without Race Concordance, Overall and by Age

Outcome	Overall PACIC^b^		Goal Setting/Tailoring		Problem-Solving/Contextual	
Model	RR^c^ (95% CI^a^)	*p*-value	RR^c^ (95% CI^a^)	*p*-value	RR^c^ (95% CI^a^)	*p*-value
Overall [*n* = 391]						
Crude	1.28 (1.05–1.56)	0.02	1.30 (1.08–1.57)	0.01	1.31 (1.09–1.57)	< 0.01
Age, gender, education adjusted	1.29 (1.07–1.56)	0.01	1.34 (1.12–1.61)	< 0.01	1.36 (1.14–1.62)	< 0.01
Age < 60 [*N* = 205]						
Crude	1.09 (0.81–1.46)	0.57	1.09 (0.84–1.42)	0.52	1.08 (0.83–1.41)	0.57
Gender + education adjusted	1.08 (0.81–1.43)	0.62	1.10 (0.86–1.42)	0.44	1.12 (0.87–1.44)	0.38
Age ≥ 60 [*N* = 186]						
Crude	1.49 (1.13–1.97)	< 0.01	1.60 (1.21–2.12)	< 0.01	1.59 (1.22–2.08)	< 0.01
Gender + education adjusted	1.53 (1.17–2.00)	< 0.01	1.63 (1.24–2.15)	< 0.01	1.66 (1.29–2.14)	< 0.01

^a^*CI*, confidence interval; ^b^*PACIC*, Patient Assessment of Chronic Illness Care; ^c^*RR*, risk ratio

See Supplemental Table 2 for complete RRs for non-significant PACIC subscales

### PACIC Subscales

A larger proportion of race concordant patients reported better goal setting/tailoring (60.9% vs 46.8%, *p* < 0.05) and problem-solving/contextualization (62.7% vs 48%, *p* < 0.05) compared to discordant pairs. Greater proportions of patients in racially concordant pairs reported better patient activation (62.3% vs 53.8%, *p* > 0.05) and follow-up/coordination (62.4% vs 53.4%, *p* > 0.05), but these differences were not significant (Fig. 1). Patients in racially concordant pairs were more likely to report better goal setting/tailoring of care (fully adjusted RR 1.34, 95% CI 1.12–1.61, *p* = 0.002) and better problem-solving/and contextual help (fully adjusted RR 1.36, 95% CI 1.14–1.62, *p* = 0.001) in both unadjusted and adjusted models (Table 2).

### Age-Related Differences

The interaction term for age and race concordance had *p* = 0.12 in the overall model, supporting the analysis stratified by age < 60 and age ≥ 60 years. There were no significant differences in the overall PACIC scores or any of the subscales for those age < 60 years, although the overall score and all but one subscale was non-significantly higher for those with race concordance (Fig. 1). However, in the older group, compared to patients with race discordance, patients with race concordance had better overall PACIC scores (65.2% vs. 43.6%, *p* < 0.05) and on the goal setting/tailoring (65.2% vs. 40.7%, *p* < 0.05) and problem-solving/contextual care subscales (69.1% vs. 43.4%, *p* < 0.05). These differences by age were also apparent in the crude and adjusted models, in which there were no significant findings for patients age < 60 years. However, among patients age ≥ 60 years, in both the overall PACIC score (fully adjusted RR 1.53, 95% CI 1.17–2.00) and the goal setting/tailoring (fully adjusted RR 1.63, 95% CI 1.24–2.15) and problem-solving/contextual subscales (fully adjusted RR 1.66, 95% CI 1.29–2.14), patients in race concordant pairs were more likely to score higher than those in race discordant pairs in both crude and adjusted analyses (Table 2).

## DISCUSSION

Our results demonstrate that, compared with Black patients in racially discordant pairs, Black patients who considered their primary care doctor to be racially concordant (i.e., also Black) perceived better chronic illness care as reflected in higher PACIC scores. Stratified analyses revealed that these findings were observed only in patients aged 60 years and older, and not in younger patients. Studies have demonstrated that higher PACIC scores (which indicate better CCM aligned care) are associated with increased self-management behaviors such as medication adherence and improved health literacy, which could lead to improved outcomes; thus, these findings have implications for efforts to eliminate health inequities.^14,15^

Our study showed associations between race concordance and PACIC subscales. *Goal Setting* and *Problem-Solving* were significantly associated with race concordance in adjusted models. Both *Goal Setting* and *Problem-Solving* subscales asked questions related to communication between patient and provider (see suppl. item 1). This result suggests that race concordance could be associated with better communication between patients and providers such that patients are able to advocate for their needs, learn how their habits affect their health, and develop a more therapeutic relationship with their provider.

An important finding in our study was that race concordance and its significant relationship with PACIC scores were only evident in patients who were age ≥ 60 years. Although our study could not explain this finding, it may be due to the lived experiences of this older generation. They were born into, grew up during, and were the leaders of the Civil Rights movement in the South, a period in American history when Black people were protesting and fighting for equality and the abolishment of Jim Crow laws.^28^ Though it continues to this day, this generation witnessed the murder of innocent Black people and Black leaders by White perpetrators who were largely unpunished until years later, if at all.^29^ The impact of such experiences may still be informing the way that Black individuals interact with the healthcare delivery system and its providers, which continues to include a smaller proportion of Black physicians than in the general population.^30^

This study adds to the body of literature supporting the need for diversity within the medical field. A randomized controlled study conducted by Alsan et al. in Oakland, CA, demonstrated that race concordance among Black men and their primary care providers led to them raising more issues during their visits and seeking more advice, indicating a positive influence on patient engagement. Alsan et al. hypothesized that these findings were possibly due to better communication in race concordant pairs.^7^ Our findings support the findings of that study, because both subscales that drove the overall findings—*Goal Setting* and *Problem-Solving*—each require good communication.

It should be noted that, while diversifying the medical field is one possible way to mitigate these disparities, there are several other contributing factors to these issues. One significant barrier is the provider shortage in areas that have a low population density, and/or are socioeconomically disadvantaged. These areas have extremely limited access to healthcare providers, and even lower access to Black providers.^31^

This study has several strengths including its population of southern Black patients living with a chronic illness, a rarely studied group. Validated scales were used where available. Limitations include its modest sample size, the impact of recall bias given that patients were asked to remember their most recent clinician experience which could have been several months ago, and potentially limited generalizability due to the unique nature of the population we sampled. Additionally, as this was an observational study, we are unable to draw causal conclusions and we acknowledge that unmeasured factors could have contributed to the relationships we observed. Lastly, because the greater SEC study was a randomized controlled trial which tested two interventions to improve BP control, we were unable to draw conclusions about race concordance and clinical outcomes as it would be difficult to determine if differences in outcomes were a result of the intervention or race concordance. Future studies should evaluate the specific contribution of patient-provider communication to PACIC scores in racially concordant and discordant pairings as well as how communication in these pairings contributes to trust in one’s provider, a precursor for changing behaviors, in additional populations.

In conclusion, in this study of Southern Black patients living with a chronic illness, race concordance between older patients and their primary care provider was associated with patient perceived higher quality of chronic illness care as measured by the PACIC. The significant subscales that drove the findings may relate to perceptions of better communication between providers and patients, although our findings should be confirmed in future studies. This study supports the importance of continued efforts to diversify the medical workforce as part of the strategy to promote equity in healthcare.

## Supplementary Information

Below is the link to the electronic supplementary material.

Supplementary file1 (DOCX 19 KB)

## Data Availability

Data are available upon request from Dr. Safford.
