# Supplementary material for: Patient Perceptions of Provider Race Concordance and Quality of Chronic Illness Care
Source: J Gen Intern Med. 2025 Jan 22;40(10):2218–25. doi: 10.1007/s11606-024-09025-w (PMC12344043; doi:10.1007/s11606-024-09025-w)
Supplement: Supplementary file 1 — Supplementary file1 (DOCX 19 KB) [file 11606_2024_9025_MOESM1_ESM.docx]

**Patient Perceptions of Provider Race Concordance and Quality of Chronic Illness Care**

Benjamin Grant, Orysya Soroka, Elizabeth Baquero, Joanna Bryan Ringel, Andrea Cherrington, Doyle M. Cummings, Jacqueline R. Halladay, Arvind Rajan, Monika M. Safford

**Supplemental Materials**

**Supplemental Item 1. Patient Assessment of Care for Chronic Conditions (PACIC) questions.**

Staying healthy can be difficult when you have a chronic illness like high blood pressure. We would like to learn about the type of help with your condition you get from your health care team. This might include your regular doctor, his or her nurse, a nurse practitioner, or a or physician’s assistant who treats your high blood pressure. Your answers will be kept confidential and will not be shared with anyone else. Think about the health care you’ve received for your high blood pressure over the past 6 months. (If it’s been more than 6 months since you’ve seen your doctor or nurse, think about your most recent visit.) Patient Response: 0=None of the time|1=A little of the time|2=Some of the time|3=Most of the time|4=Always

| **Patient Activation** |
| --- |
| Asked for your ideas when making a treatment plan? |
| Given choices about treatment to think about? |
| Asked to talk about any problems with your medicines or their effects? |
| **Delivery System Design/Decision Support** |
| Given a written list of things you should do to improve your health? |
| Satisfied that your care was well organized? |
| Shown how what you did to take care of yourself influenced your high blood pressure? |
| **Goal Setting/Tailoring** |
| Asked to talk about your goals in caring for your high blood pressure? |
| Helped to set specific goals to improve your eating or exercise? |
| Given a copy of your treatment plan? |
| Encouraged to go to a specific group or class to help you cope with your high blood pressure? |
| Asked questions, either directly or on a survey, about your health habits? |
| **Problem-Solving/Contextual** |
| Sure, that your doctor/nurse thought about your values, beliefs, & traditions when they recommended treatments to you? |
| Helped to make a treatment plan that you could carry out in your daily life? |
| Helped to plan so you could take care of your high blood pressure even in hard times? |
| Asked how your high blood pressure affects your life? |
| **Follow-up/Coordination** |
| Contacted after a visit to see how things were going? |
| Encouraged to attend programs in the community that could help you? |
| Referred to a dietitian, health educator, or counselor? |
| Told how your visits with other types of doctors, like an eye doctor or other specialists, helped your treatment? |
| Asked how your visits with other doctors were going? |

**Suppl. Table 1: Risk Ratios (95% CI^1^) for having higher PACIC^2^ subscale scores in patients with patient-provider race concordance versus patients without race concordance, overall and by age, for nonsignificant subscales.**

| **Outcome** | **Patient Activation** | | | **Delivery System Design/Decision Support** | | **Follow-up/ Coordination** | | |
| --- | --- | --- | --- | --- | --- | --- | --- | --- |
| **Model** | **RR^3^**  **(95% CI^1^)** | | **p** | **RR^3^**  **(95% CI^1^)** | **p** | **RR^3^**  **(95% CI^1^)** | | **p** |
| **Overall [n=391]** |  |  | | | |  |  | |
| Crude | 1.16  (0.97-1.38) | | 0.10 | 1.00  (0.79-1.26) | 0.99 | 1.17  (0.98-1.39) | | 0.08 |
| Age, gender, + education adjusted | 1.16  (0.97-1.37) | | 0.10 | 1.00  (0.80-1.25) | 0.99 | 1.14  (0.96-1.36) | | 0.13 |
| **Age <60 [N=205].** |  |  | | | |  |  | |
| Crude | 1.18  (0.93-1.50) | | 0.18 | 0.76  (0.53-1.10) | 0.15 | 1.14  (0.89-1.46) | | 0.30 |
| Gender + education adjusted | 1.18  (0.93-1.50) | | 0.17 | 0.76  (0.53-1.08) | 0.12 | 1.10  (0.86-1.42) | | 0.44 |
| **Age ≥60 [N=186]** |  |  | | | |  |  | |
| Crude | 1.14  (0.89-1.46) | | 0.31 | 1.28  (0.93-1.74) | 0.13 | 1.19  (0.94-1.51) | | 0.15 |
| Gender + education adjusted | 1.14  (0.89-1.46) | | 0.32 | 1.27  (0.93-1.74) | 0.13 | 1.18  (0.93-1.50) | | 0.17 |

*Abbreviations*: ^1^CI, Confidence Interval; ^2^PACIC, Patient Assessment of Care for Chronic Conditions; ^3^RR, Risk Ratio.
